# Family history of cancer and risk of lung cancer: a retrospective cohort study in a Chinese population

**DOI:** 10.3389/fmed.2026.1918599

**Published:** 2026-08-24

**Authors:** Hongjun Song, Xinlu Men, Jun Cheng, Liangliang Zhang, Shuang Zhao

**Affiliations:** 1 Outpatient Department, West China School of Nursing, West China Hospital, Sichuan University, Chengdu, Sichuan, China; 2 Outpatient Department, West China Hospital, Sichuan University, Chengdu, Sichuan, China; 3 Department of Anesthesiology, West China Hospital, Sichuan University, Chengdu, Sichuan, China; 4 Department of Anesthesiology, West China School of Nursing, Sichuan University, Chengdu, Sichuan, China; 5 Department of Pulmonary and Critical Care Medicine, West China Hospital, Sichuan University, Chengdu, Sichuan, China; 6 State Key Laboratory of Respiratory Health and Multimorbidity, Department of Respiratory and Critical Care Medicine, West China Hospital, Sichuan University, Chengdu, Sichuan, China

**Keywords:** Chinese population, family history, genetic predisposition, lung cancer, polygenic risk score, pulmonary nodule

## Abstract

**Background:**

Family history of cancer is an established risk factor for lung cancer, but its association with the probability of malignancy among patients evaluated for pulmonary nodules—rather than with population-level lung cancer incidence—remains poorly characterized in Chinese populations.

**Methods:**

We conducted a retrospective cohort study of 3,850 consecutive patients evaluated for pulmonary nodules at a tertiary teaching hospital between January 2018 and December 2021, of whom 1,257 (32.6%) underwent surgical resection or biopsy. Family history of lung and non-lung cancer in first- and second-degree relatives was self-reported at admission. Lung cancer was confirmed histopathologically. Sequential multivariable logistic regression was performed: Model 1 included clinical variables only, and Model 2 additionally included radiological features.

**Results:**

Among 3,850 patients, 1,158 (30.1%) had pathology-confirmed lung cancer (95.3% adenocarcinoma; 92.2% stage IA). The 2,692 controls comprised 99 patients with pathology-confirmed benign lesions and 2,593 patients managed with imaging surveillance in whom no malignant pathology was recorded. First-degree family history of lung cancer (present in 32.3% of the cohort) was not associated with malignancy in univariate analysis (OR 0.94, 95% CI 0.81–1.09, *p* = 0.432) but showed an inverse association after adjustment (Model 2: aOR 0.72, 95% CI 0.57–0.90, *p* = 0.005); family history of non-lung cancer showed a similar pattern (univariate OR 0.79, 95% CI 0.69–0.91; Model 2: aOR 0.59, 95% CI 0.48–0.74, *p* < 0.001). Radiological features dominated risk prediction (Model 2 AUC 0.807 versus 0.566 for clinical variables alone): nodule size (aOR 1.21 per mm, 95% CI 1.18–1.24), lobulation (aOR 2.32), spiculation (aOR 2.08), and vacuole sign (aOR 1.80) were the strongest predictors. Female sex was independently associated with malignancy (aOR 1.62, 95% CI 1.31–2.01), whereas smoking was not (aOR 1.16, 95% CI 0.90–1.49). Patients with family history were not diagnosed earlier or at an earlier stage than those without.

**Conclusion:**

In this selected cohort of patients evaluated for pulmonary nodules, family history was not positively associated with malignancy, and the inverse adjusted associations are most plausibly explained by referral- and surveillance-related selection rather than biological protection. Radiological assessment should remain the primary basis for pulmonary nodule management. Family history may be considered during nodule evaluation, but its independent contribution should be validated in prospective cohorts with standardized screening and referral procedures.

## 1 Introduction

Lung cancer is the most common cause of cancer-related mortality globally, accounting for approximately 1.8 million deaths annually (1). In China, lung cancer incidence and mortality rates have been rising steadily, with an estimated 815,000 new cases and 714,000 deaths in 2020 (2). While tobacco smoking remains the primary risk factor, approximately 15–25% of lung cancer cases occur in never-smokers, particularly in Asian populations (3). This phenomenon has prompted increased interest in genetic susceptibility, familial aggregation, and gene–environment interactions in lung cancer etiology.

Family history of cancer is a well-established risk factor for lung cancer: individuals with a first-degree relative diagnosed with lung cancer have a 1.5- to 2-fold elevated disease risk (4, 5). The genetic component of lung cancer susceptibility is supported by genome-wide association studies (GWAS) that have identified multiple susceptibility loci, including variants at 15q25 (CHRNA3–CHRNA5 nicotinic acetylcholine receptor subunits), 5p15.33 (TERT–CLPTM1L), and 6p21 (BAT3–MSH5) (6, 7). However, the majority of lung cancer cases cannot be explained by single genetic variants, suggesting a complex interplay between genetic predisposition and environmental exposures.

Recent advances in next-generation sequencing have revealed that 3.5–21.4% of lung cancer patients harbor pathogenic or likely pathogenic germline variants in cancer predisposition genes, with ATM, BRCA2, TP53, and CHEK2 being the most frequently implicated (8, 9). These germline alterations are more prevalent in younger patients, females, and never-smokers, underscoring the importance of genetic susceptibility in lung cancer development (8). Furthermore, familial aggregation studies have demonstrated that lung cancer risk is elevated not only among first-degree relatives but also among second- and third-degree relatives, suggesting a meaningful genetic contribution beyond shared household environments (10).

The role of family history of non-lung cancer in lung cancer risk is less well characterized. Shared genetic susceptibility across cancer types, particularly genes related to DNA repair (e.g., BRCA1, BRCA2, ATM, and CHEK2), cell cycle regulation (e.g., TP53 and RB1), and carcinogen metabolism (e.g., CYP1A1 and GSTM1), may contribute to increased lung cancer risk in individuals with a family history of other malignancies (11). Additionally, shared environmental exposures within families, including secondhand smoke, indoor air pollution from cooking, and occupational hazards, may confound the association between family history and lung cancer risk (12).

With the widespread use of chest computed tomography (CT) in China, an increasing number of patients are referred for evaluation of pulmonary nodules. In this setting, the clinically relevant question is not whether family history influences population-level lung cancer incidence, but whether it is associated with the probability that a detected nodule is malignant. The present study therefore evaluated the association between family history of cancer (both lung and non-lung) and the probability of malignancy in a large cohort of Chinese patients evaluated for pulmonary nodules, using sequential multivariable models that separately consider clinical and radiological factors.

## 2 Materials and methods

### 2.1 Study design and population

This was a retrospective cohort study conducted at West China Hospital, Sichuan University, a tertiary teaching hospital in China. Consecutive patients who were evaluated for pulmonary nodules between January 2018 and December 2021 were included. Importantly, this was a clinic-based cohort assembled from outpatient consultations, health check-up referrals, and inpatient work-up; it was not drawn from the general population or from a standardized lung cancer screening program. Consequently, the study evaluates the probability of malignancy among patients with pulmonary nodules under clinical evaluation, rather than the population-level risk of developing lung cancer.

Of the 3,850 included patients, 1,257 (32.6%) underwent surgical resection or biopsy. Cases (*n* = 1,158) were patients with histopathologically confirmed primary lung cancer. The control group (*n* = 2,692) comprised 99 patients with pathology-confirmed benign lesions and 2,593 patients managed with imaging surveillance in whom no malignant pathology was recorded during the available follow-up period. Patients with incomplete family history documentation or missing outcome data were excluded; all 3,850 patients in the analytic cohort had complete data for the variables used in the analyses, and no imputation was required. The study was approved by the West China Hospital, Sichuan University Clinical Research Ethics Committee (approval no. 2020–232), and the requirement for individual informed consent was waived because of the retrospective design.

### 2.2 Data collection

Clinical, demographic, radiological, and environmental exposure data were extracted from electronic medical records. The following variables were collected: demographics (age, sex, date of specimen collection); smoking history (smoking status, cigarettes per day, smoking duration, years since quitting, pack-years); family history of cancer (see Section 2.3); environmental and occupational exposures (occupational exposure to carcinogens); comorbidities (chronic obstructive pulmonary disease [COPD], pulmonary fibrosis, tuberculosis, asthma); radiological features (nodule location, nodule type [pure ground-glass opacity (pGGO), mixed ground-glass opacity (mGGO), solid], nodule size [maximum diameter in mm], lobulation, spiculation, vacuole sign, pleural indentation); and pathology (histological type, TNM stage [8th edition]).

### 2.3 Family history assessment

Family history was self-reported by patients at the time of admission and recorded in the electronic medical record using a structured history-taking template; it was not verified against cancer registries. First-degree relatives (FDRs) were defined as parents, siblings, and children; second-degree relatives were defined as grandparents, grandchildren, uncles/aunts, and nephews/nieces. Family history of lung cancer was defined as at least one relative with lung cancer and was recorded separately for first-degree versus non-first-degree relatives. Family history of non-lung cancer was defined as at least one relative with any non-lung malignancy. Patients with multiple affected relatives were counted once within each applicable category. Patients reporting both lung cancer and non-lung cancer family histories were included in both variables; the two family-history variables are therefore not mutually exclusive. For the distribution of family cancer types (Table 1), each reported malignancy in a relative was counted as one event, such that a patient with two or more affected relatives could contribute multiple events; non-malignant conditions recorded in the same free-text fields (e.g., hypertension, diabetes) were excluded from this analysis.

**Table 1:** Distribution of family cancer types among relatives of lung cancer patients (1,039 events reported by 1,158 patients).

Cancer type in relative	Events, *n*	% of all events
Lung cancer	400	38.5%
Liver cancer	105	10.1%
Esophageal cancer	84	8.1%
Rectal cancer	77	7.4%
Gastric cancer	72	6.9%
Pancreatic cancer	43	4.1%
Breast cancer	40	3.8%
Colon cancer	35	3.4%
Cervical cancer	22	2.1%
Nasopharyngeal cancer	20	1.9%
Bladder cancer	17	1.6%
Lymphoma	17	1.6%
Prostate cancer	14	1.3%
Thyroid cancer	13	1.3%
Colorectal cancer, unspecified	13	1.3%
Leukemia	10	1.0%
Laryngeal cancer	10	1.0%
Oral cancer	10	1.0%
Bile duct cancer	10	1.0%
Uterine cancer	8	0.8%
Kidney cancer	5	0.5%
Tongue cancer	5	0.5%
Brain tumor	5	0.5%
Gallbladder cancer	4	0.4%

Numbers represent reported cancer events among relatives, not patients; patients with multiple affected relatives could contribute to multiple events. Non-malignant conditions recorded in the same fields were excluded.

### 2.4 Definitions

Lung cancer was defined as any malignant pulmonary neoplasm confirmed by histopathological examination following surgical resection or biopsy. Pathological categories (lung adenocarcinoma, minimally invasive adenocarcinoma, adenocarcinoma *in situ*, invasive adenocarcinoma, and other histologies) were mutually exclusive and were assigned from the final histopathology report according to the 2021 World Health Organization classification of lung tumors; where reports used generic terminology (e.g., “lung adenocarcinoma”) without further subclassification, the generic category was retained. Benign lesions included inflammatory nodules, granulomas, hamartomas, and other non-malignant pathologies. Ever smoking was defined as smoking at least 100 cigarettes in a lifetime, and heavy smoking as ≥20 pack-years.

### 2.5 Statistical analysis

Continuous variables were presented as mean ± standard deviation (SD) and compared using Welch’s *t*-test. Categorical variables were presented as frequencies and percentages and compared using chi-square tests or Fisher’s exact tests, as appropriate. Univariate logistic regression was used to calculate crude odds ratios (ORs) and 95% confidence intervals (CIs).

Multivariable logistic regression was performed sequentially to make the adjustment process transparent. Model 1 included clinical variables only (age, sex, smoking status, family history of lung cancer, family history of non-lung cancer, COPD, and occupational exposure). Model 2 additionally included radiological variables (nodule size, nodule type, lobulation, spiculation, vacuole sign, and pleural indentation). Variables were selected *a priori* on the basis of clinical relevance and established nodule risk models. Model discrimination was assessed using the area under the receiver operating characteristic curve (AUC).

Stratified analyses were performed by smoking status (never vs. ever), sex, and age group (<50 vs. ≥50 years), and multiplicative interaction terms were tested in the multivariable model. For the time-to-diagnosis analysis, the interval (in years) from first CT detection of the nodule to pathological diagnosis was calculated among lung cancer cases; distributions were compared between patients with and without family history of lung cancer using the Mann–Whitney U test, and cumulative diagnosis proportions were plotted over follow-up time. The proportion of stage IA disease was compared using the chi-square test. Statistical significance was defined as a two-sided *p*-value <0.05. All analyses were performed using Python 3.

## 3 Results

### 3.1 Patient characteristics

**Figure 1:**
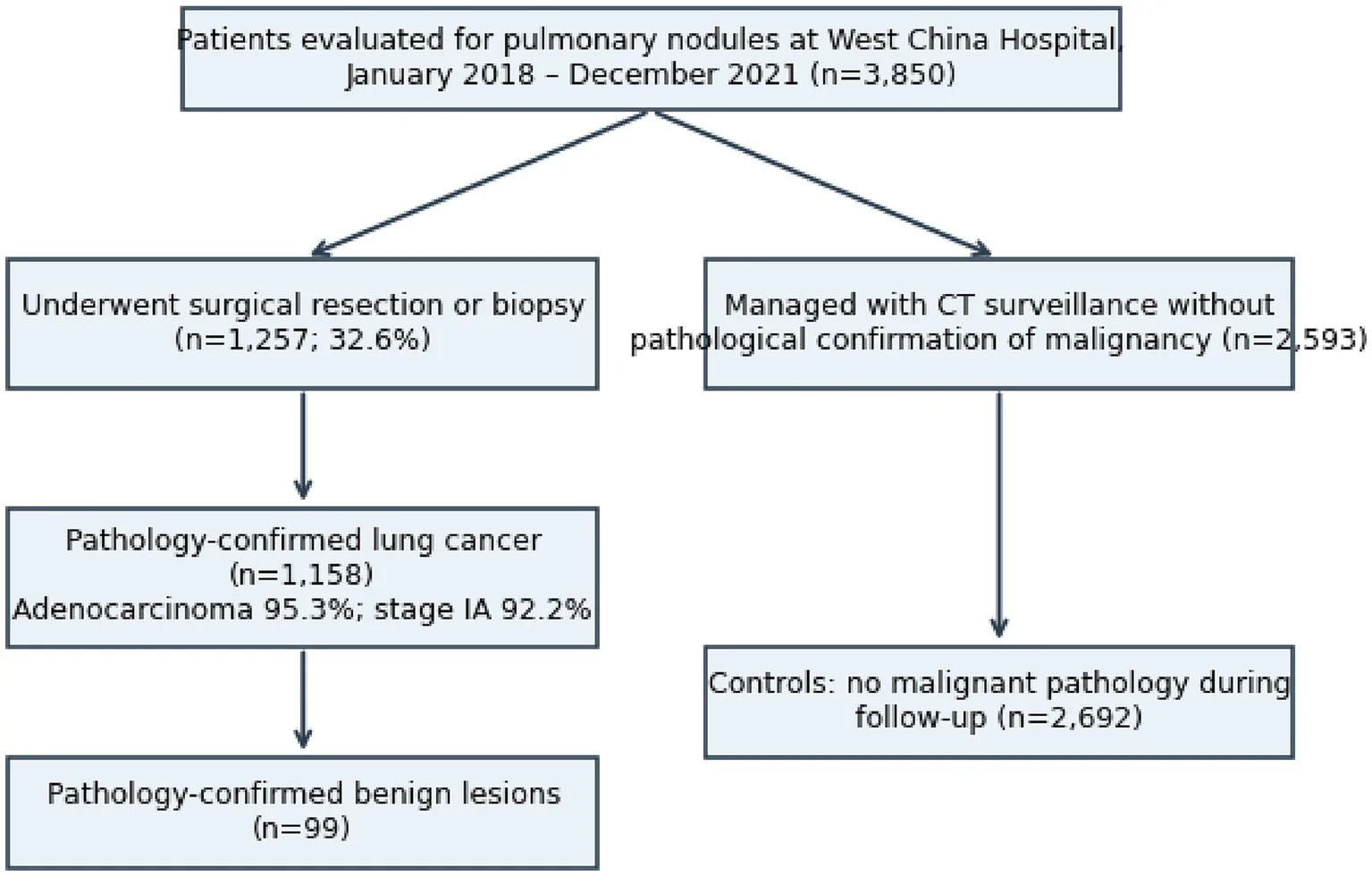
Flow chart of included patients.

A total of 3,850 patients evaluated for pulmonary nodules were included (Figure 1), of whom 1,158 (30.1%) had pathology-confirmed lung cancer and 2,692 (69.9%) served as controls. The mean age was 49.5 ± 11.8 years (range 15–84 years), and 2,548 patients (66.2%) were female; 79.9% of the cohort were never-smokers. Among lung cancer patients, 95.3% had adenocarcinoma and 92.2% were diagnosed at stage IA (Tables 2, 3).

**Table 2:** Pathological distribution among lung cancer patients (*n* = 1,158).

Pathology	Count	Percentage
Lung adenocarcinoma	1,103	95.3%
Minimally invasive adenocarcinoma	21	1.8%
Adenocarcinoma in situ	10	0.9%
Invasive adenocarcinoma	5	0.4%
Pulmonary lymphoma	5	0.4%
Lung squamous cell carcinoma	3	0.3%
Invasive non-mucinous adenocarcinoma	2	0.2%
Metastatic/secondary malignancy	2	0.2%
Neuroendocrine tumor/carcinoid	2	0.2%
Small cell lung cancer	1	0.1%
Adenosquamous carcinoma	1	0.1%
Other/unspecified	3	0.3%

Categories were mutually exclusive and assigned from the final histopathology report according to the 2021 WHO classification; generic terminology was retained where reports did not further subclassify the tumor.

**Table 3:** TNM stage distribution among lung cancer patients (*n* = 1,158).

Stage (8th edition)	Count	Percentage
Stage IA (IA1 676; IA2 340; IA3 52)	1,068	92.2%
Stage IB	61	5.3%
Stage II	3	0.3%
Stage III	7	0.6%
Stage IV	8	0.7%
Unknown	11	0.9%

Baseline characteristics are presented in Table 4. Lung cancer patients were slightly older than controls (50.2 ± 11.7 vs. 49.2 ± 11.8 years, *p* = 0.008), were more frequently female (71.3% vs. 64.0%, *p* < 0.001), and had larger nodules (11.0 ± 5.3 vs. 7.4 ± 3.6 mm, *p* < 0.001). Mixed ground-glass nodules were substantially more frequent among cases (44.4% vs. 17.0%, p < 0.001), whereas pure ground-glass (48.4% vs. 58.6%, *p* < 0.001) and solid nodules (7.2% vs. 24.4%, *p* < 0.001) were less frequent. Ever-smoking did not differ significantly between groups (18.4% vs. 20.9%, *p* = 0.078). First-degree family history of lung cancer was similarly frequent in cases and controls (31.4% vs. 32.7%, *p* = 0.432), whereas family history of non-lung cancer was less frequent among cases (56.0% vs. 61.7%, *p* < 0.001). All patient counts in the text, tables, and figures were re-audited against the source dataset during revision.

**Table 4:** Baseline characteristics of lung cancer patients and controls (*n* = 3,850).

Variable	Cases (*n* = 1,158)	Controls (*n* = 2,692)	*P*-value
Age, years (mean ± SD)	50.2 ± 11.7	49.2 ± 11.8	0.008
Female, *n* (%)	826 (71.3%)	1,722 (64.0%)	<0.001
Ever smoking, *n* (%)	213 (18.4%)	562 (20.9%)	0.078
Heavy smoking (≥20 pack-years), *n* (%)	106 (9.2%)	236 (8.8%)	0.699
Family history of lung cancer, first-degree, *n* (%)	364 (31.4%)	881 (32.7%)	0.432
Family history of lung cancer, any degree, *n* (%)	485 (41.9%)	1,117 (41.5%)	0.822
Family history of non-lung cancer, *n* (%)	648 (56.0%)	1,661 (61.7%)	<0.001
COPD, *n* (%)	9 (0.8%)	9 (0.3%)	0.065
Occupational exposure, *n* (%)	11 (0.9%)	34 (1.3%)	0.407
Nodule size, mm (mean ± SD)	11.0 ± 5.3	7.4 ± 3.6	<0.001
pGGO nodule, *n* (%)	561 (48.4%)	1,577 (58.6%)	<0.001
mGGO nodule, *n* (%)	514 (44.4%)	458 (17.0%)	<0.001
Solid nodule, *n* (%)	83 (7.2%)	657 (24.4%)	<0.001

SD, standard deviation; COPD, chronic obstructive pulmonary disease; pGGO, pure ground-glass opacity; mGGO, mixed ground-glass opacity. Controls comprised 99 pathology-confirmed benign lesions and 2,593 patients under imaging surveillance without malignant pathology. Percentages for nodule type are within-group proportions.

### 3.2 Family history and lung cancer risk: univariate analysis

In univariate analysis (Table 5), first-degree family history of lung cancer was not significantly associated with the probability of malignancy (OR 0.94, 95% CI 0.81–1.09, *p* = 0.432), and non-first-degree family history showed a non-significant positive trend (OR 1.20, 95% CI 0.95–1.52, *p* = 0.131). Family history of non-lung cancer was inversely associated with malignancy (OR 0.79, 95% CI 0.69–0.91, *p* < 0.001). Neither ever-smoking (OR 0.85, 95% CI 0.72–1.02, *p* = 0.078) nor heavy smoking (OR 1.05, 95% CI 0.82–1.33, *p* = 0.697) was significantly associated with malignancy in this nodule-based cohort.

**Table 5:** Univariate analysis of family history, smoking, and probability of malignancy.

Variable	Cases/Total (%)	OR (95% CI)	*P*-value
Family history of lung cancer, first-degree	364/1,245 (29.2%)	0.94 (0.81–1.09)	0.432
No family history of lung cancer, first-degree	794/2,605 (30.5%)	1.00 (reference)	—
Family history of lung cancer, non-first-degree	121/357 (33.9%)	1.20 (0.95–1.52)	0.131
No family history of lung cancer	673/2,248 (29.9%)	1.00 (reference)	—
Family history of non-lung cancer	648/2,309 (28.1%)	0.79 (0.69–0.91)	<0.001
No family history of non-lung cancer	510/1,541 (33.1%)	1.00 (reference)	—
Ever smoking	213/775 (27.5%)	0.85 (0.72–1.02)	0.078
Never smoking	945/3,075 (30.7%)	1.00 (reference)	—
Heavy smoking (≥20 pack-years)	106/342 (31.0%)	1.05 (0.82–1.33)	0.697

For the non-first-degree comparison, the reference category comprised patients with no family history of lung cancer in any relative. Denominators are patients with complete data for each variable (no missing data).

### 3.3 Multivariable logistic regression analysis

**Figure 2:**
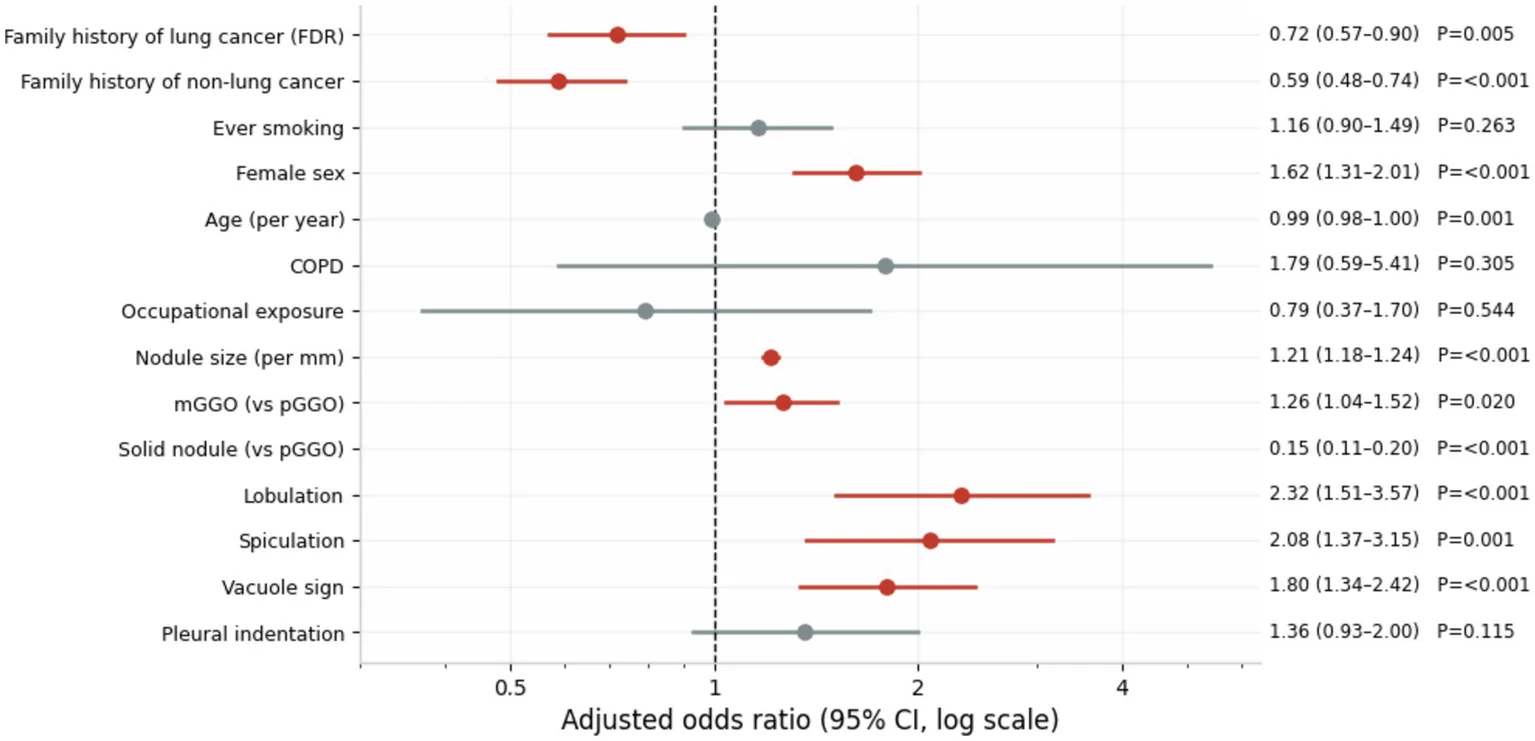
Forest plot of multivariable logistic regression (Model 2: clinical + radiological variables), *n* = 3,850.

Results of the sequential multivariable models are presented in Table 6 and Figure 2. In Model 1 (clinical variables only), first-degree family history of lung cancer (aOR 0.68, 95% CI 0.55–0.83, *p* < 0.001) and family history of non-lung cancer (aOR 0.62, 95% CI 0.51–0.75, *p* < 0.001) were inversely associated with malignancy, and female sex was positively associated (aOR 1.48, 95% CI 1.23–1.79, *p* < 0.001). In Model 2, which additionally included radiological variables, the family-history estimates changed only modestly (first-degree family history of lung cancer: aOR 0.72, 95% CI 0.57–0.90, *p* = 0.005; family history of non-lung cancer: aOR 0.59, 95% CI 0.48–0.74, *p* < 0.001), while radiological features emerged as the dominant predictors. Model discrimination improved from an AUC of 0.566 (Model 1) to 0.807 (Model 2).

**Table 6:** Sequential multivariable logistic regression for probability of malignancy (*n* = 3,850).

Variable	Model 1 (clinical): aOR (95% CI)	*P*-value	Model 2 (clinical + radiological): aOR (95% CI)	*P*-value
Family history of lung cancer, first-degree	0.68 (0.55–0.83)	<0.001	0.72 (0.57–0.90)	0.005
Family history of non-lung cancer	0.62 (0.51–0.75)	<0.001	0.59 (0.48–0.74)	<0.001
Ever smoking	1.11 (0.89–1.39)	0.338	1.16 (0.90–1.49)	0.263
Female sex	1.48 (1.23–1.79)	<0.001	1.62 (1.31–2.01)	<0.001
Age (per year)	1.01 (1.00–1.01)	0.077	0.99 (0.98–1.00)	0.001
COPD	2.19 (0.86–5.62)	0.102	1.79 (0.59–5.41)	0.305
Occupational exposure	0.81 (0.40–1.60)	0.538	0.79 (0.37–1.70)	0.544
Nodule size (per mm)	—	—	1.21 (1.18–1.24)	<0.001
mGGO (vs. pGGO)	—	—	1.26 (1.04–1.52)	0.020
Solid (vs. pGGO)	—	—	0.15 (0.11–0.20)	<0.001
Lobulation	—	—	2.32 (1.51–3.57)	<0.001
Spiculation	—	—	2.08 (1.37–3.15)	0.001
Vacuole sign	—	—	1.80 (1.34–2.42)	<0.001
Pleural indentation	—	—	1.36 (0.93–2.00)	0.115

aOR, adjusted odds ratio; CI, confidence interval; AUC, area under the receiver operating characteristic curve. Model 1 AUC = 0.566; Model 2 AUC = 0.807. All 3,850 patients had complete data and were included in both models.

To make the change from a null crude association to an inverse adjusted association transparent, we examined the estimates stepwise. Adding sex, age, and smoking status to the univariate model changed the OR for family history of lung cancer only minimally (0.94 to 0.95). The shift to an inverse association occurred when the two family-history variables were entered into the model together (OR 0.95 to 0.68): the two variables are correlated, because patients who report any family cancer history often report both lung and non-lung cancers in their relatives, and mutual adjustment separates their respective associations. Subsequent addition of radiological variables produced only small further changes (0.68 to 0.72). The inverse adjusted association therefore arises from the joint modeling of the two correlated family-history variables within this selected cohort, rather than from adjustment for imaging features. We interpret this finding cautiously as a likely consequence of selection, referral, and surveillance-related biases (see Section 4.2), not as evidence of biological protection.

### 3.4 Stratified analysis

**Figure 3:**
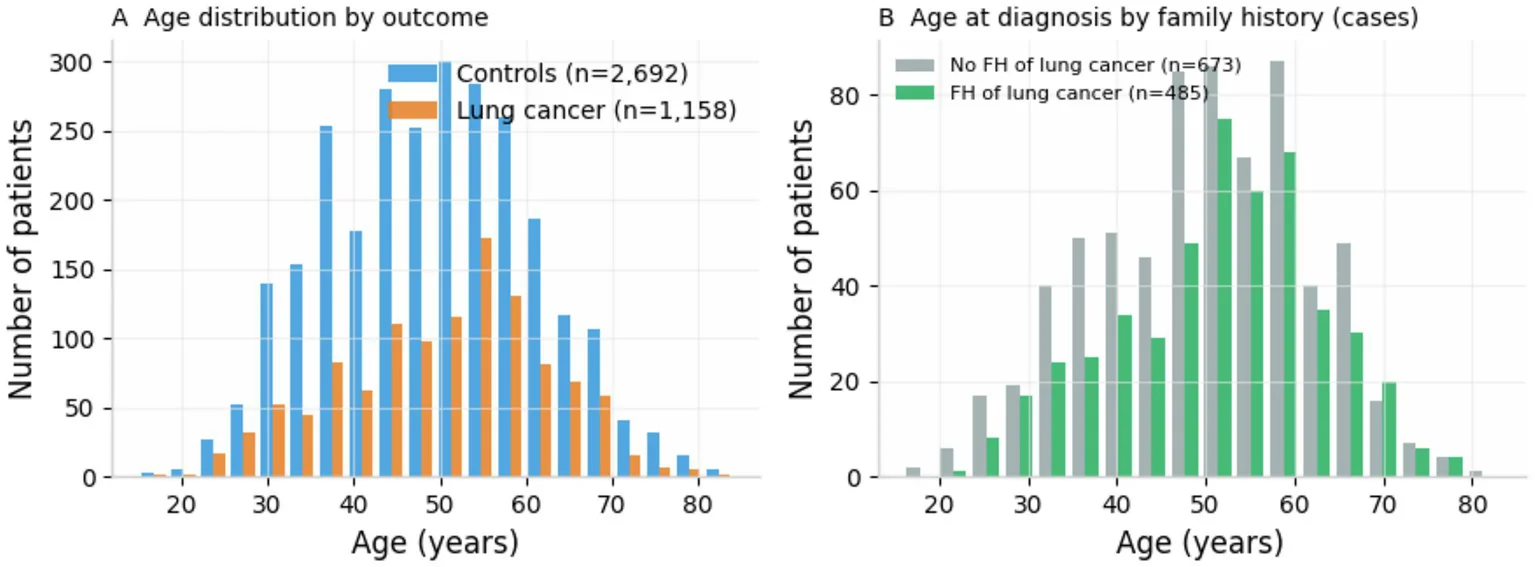
Demographic characteristics. **(A)** Age distribution by outcome; **(B)** Age at diagnosis by family history of lung cancer among cases.

In stratified analyses (Table 7), first-degree family history of lung cancer was not significantly associated with malignancy in any subgroup defined by smoking status, sex, or age, and no significant multiplicative interaction was detected (P for interaction: smoking 0.986, sex 0.305, age 0.339). All subgroup denominators sum to the full cohort of 3,850 patients; no patients were excluded from any stratum because of missing data (Figure 3).

**Table 7:** Stratified analysis: First-degree family history of lung cancer and probability of malignancy.

Stratum (n)	Cases/Total with family history (%)	Cases/Total without family history (%)	OR (95% CI)	*P*-value
Ever smokers (*n* = 775)	68/255 (26.7%)	145/520 (27.9%)	0.94 (0.67–1.32)	0.721
Never smokers (*n* = 3,075)	296/990 (29.9%)	649/2,085 (31.1%)	0.94 (0.80–1.11)	0.490
Female (*n* = 2,548)	268/831 (32.3%)	558/1,717 (32.5%)	0.99 (0.83–1.18)	0.900
Male (*n* = 1,302)	96/414 (23.2%)	236/888 (26.6%)	0.83 (0.64–1.10)	0.191
Age <50 years (*n* = 1,848)	167/650 (25.7%)	336/1,198 (28.0%)	0.89 (0.71–1.10)	0.278
Age ≥50 years (*n* = 2,002)	197/595 (33.1%)	458/1,407 (32.6%)	1.03 (0.84–1.26)	0.808

Stratum denominators sum to the full cohort (3,850); no missing data. P for interaction: smoking status 0.986, sex 0.305, age group 0.339.

### 3.5 Nodule characteristics and family history

**Figure 4:**
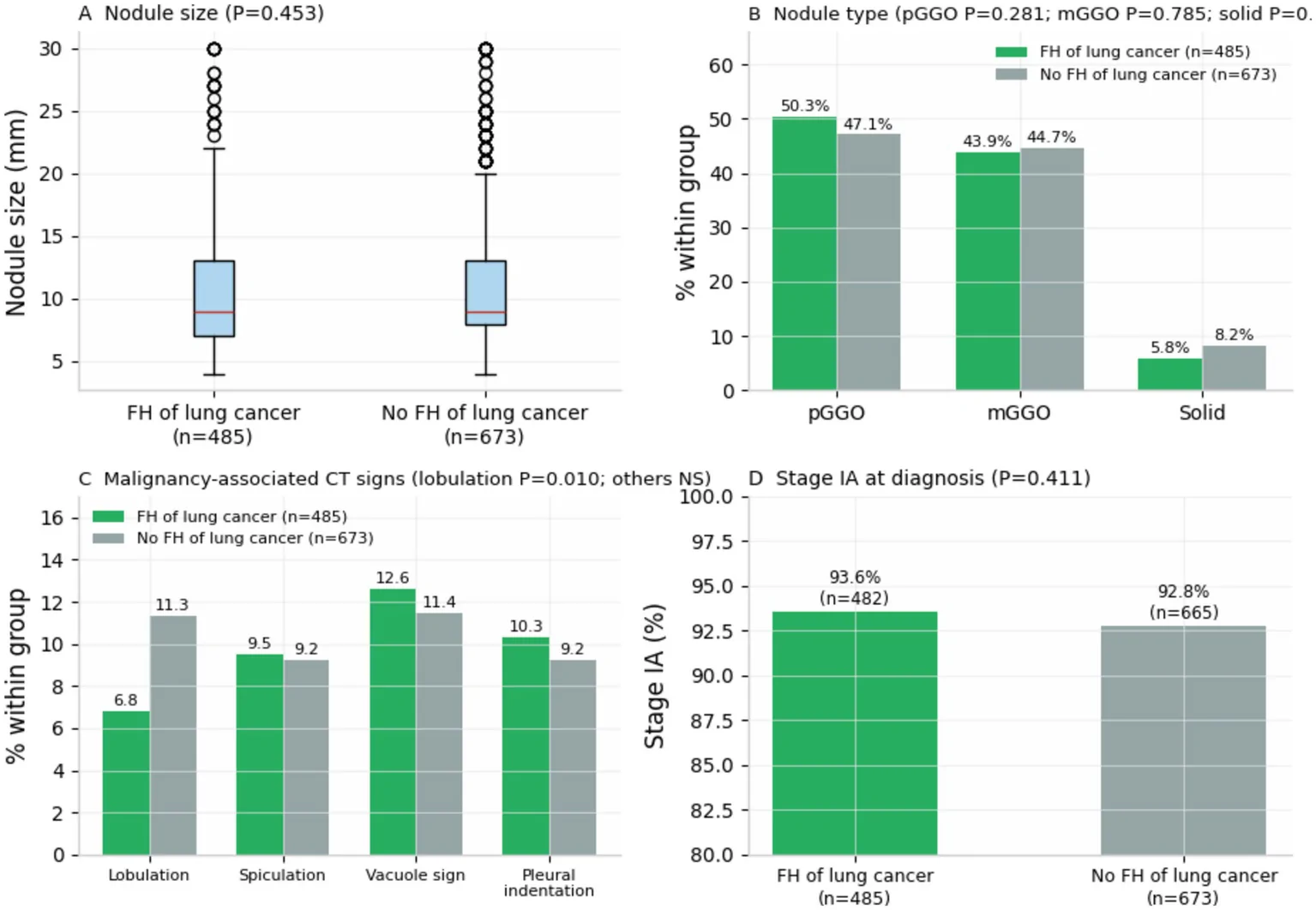
Nodule characteristics and family history of lung cancer among lung cancer patients (*n* = 1,158). **(A)** Nodule size; **(B)** Nodule type; **(C)** Malignancy-associated CT signs; **(D)** Stage IA at diagnosis.

Among lung cancer patients (Figure 4), those with a family history of lung cancer did not differ significantly from those without in nodule size (10.9 ± 5.1 vs. 11.1 ± 5.4 mm, *p* = 0.453) or nodule type distribution (pGGO 50.3% vs. 47.1%, *p* = 0.281; mGGO 43.9% vs. 44.7%, *p* = 0.785; solid 5.8% vs. 8.2%, *p* = 0.118). Lobulation was less frequent among patients with family history (6.8% vs. 11.3%, *p* = 0.010), whereas spiculation (9.5% vs. 9.2%, *p* = 0.875), vacuole sign (12.6% vs. 11.4%, *p* = 0.556), and pleural indentation (10.3% vs. 9.2%, *p* = 0.533) did not differ significantly. Patients with family history were slightly older at diagnosis (51.3 ± 11.4 vs. 49.5 ± 11.9 years, *p* = 0.013).

### 3.6 Family cancer types among lung cancer patients

**Figure 5:**
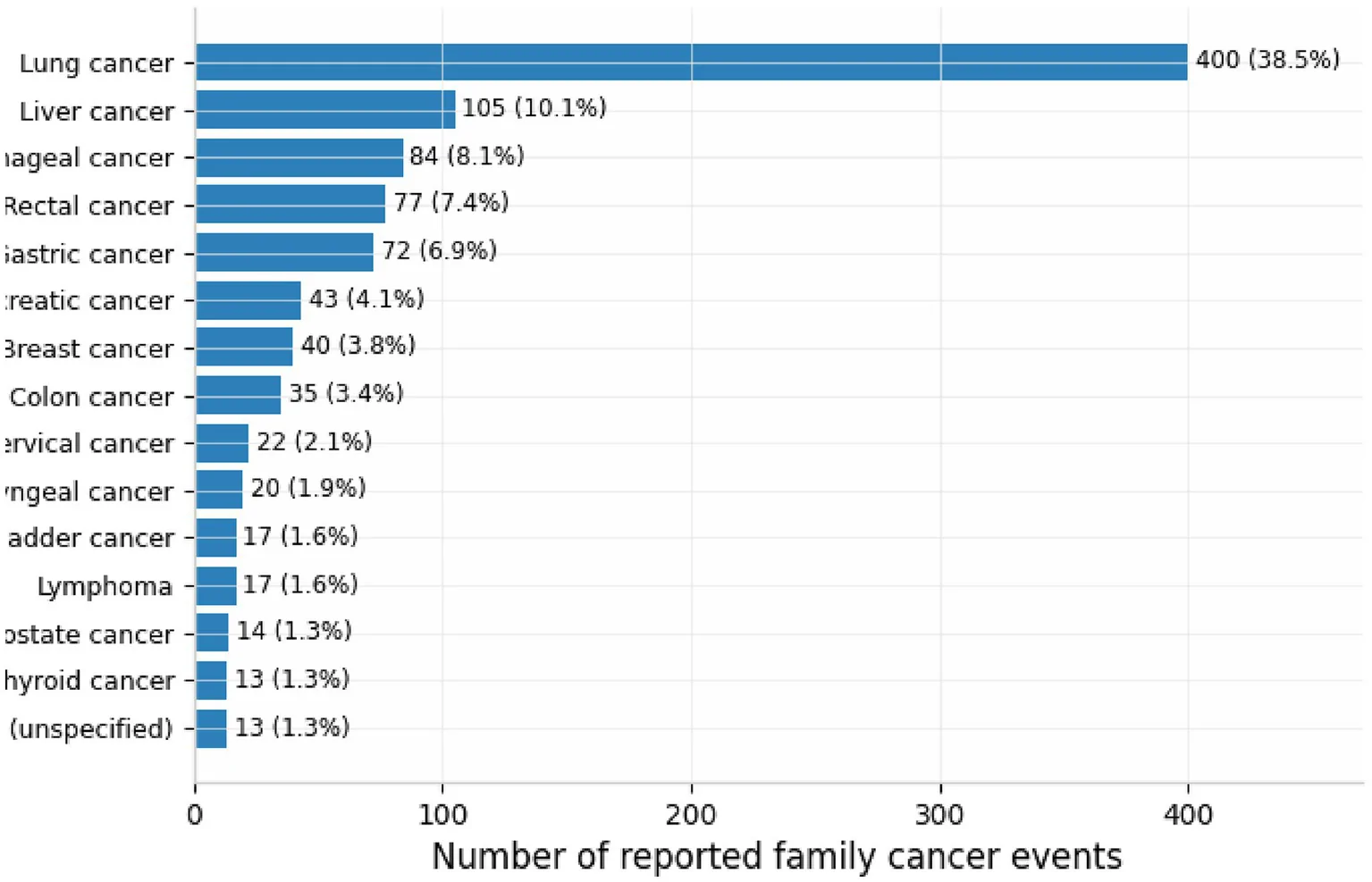
Distribution of family cancer types among relatives of lung cancer patients (1,039 cancer events reported by 1,158 patients; multiple events per patient possible).

The 1,158 lung cancer patients reported a total of 1,039 cancer events among their relatives (Table 1, Figure 5); because patients could report multiple affected relatives and multiple cancer types, the numbers represent cancer events, not individual patients. Lung cancer was the most frequently reported family cancer (400 events, 38.5%), followed by liver cancer (105, 10.1%), esophageal cancer (84, 8.1%), rectal cancer (77, 7.4%), gastric cancer (72, 6.9%), pancreatic cancer (43, 4.1%), and breast cancer (40, 3.8%). Of the 485 patients with a first- or second-degree family history of lung cancer, 392 had a corresponding lung cancer event recorded in the disease-detail fields; in the remainder, the family history was captured in the structured family-history field without a fully specified cancer type in the free-text disease fields. The clustering of gastrointestinal cancers (esophageal, gastric, colorectal, pancreatic) is consistent with shared environmental exposures in Chinese populations (13).

### 3.7 Time to diagnosis and stage at diagnosis

**Figure 6:**
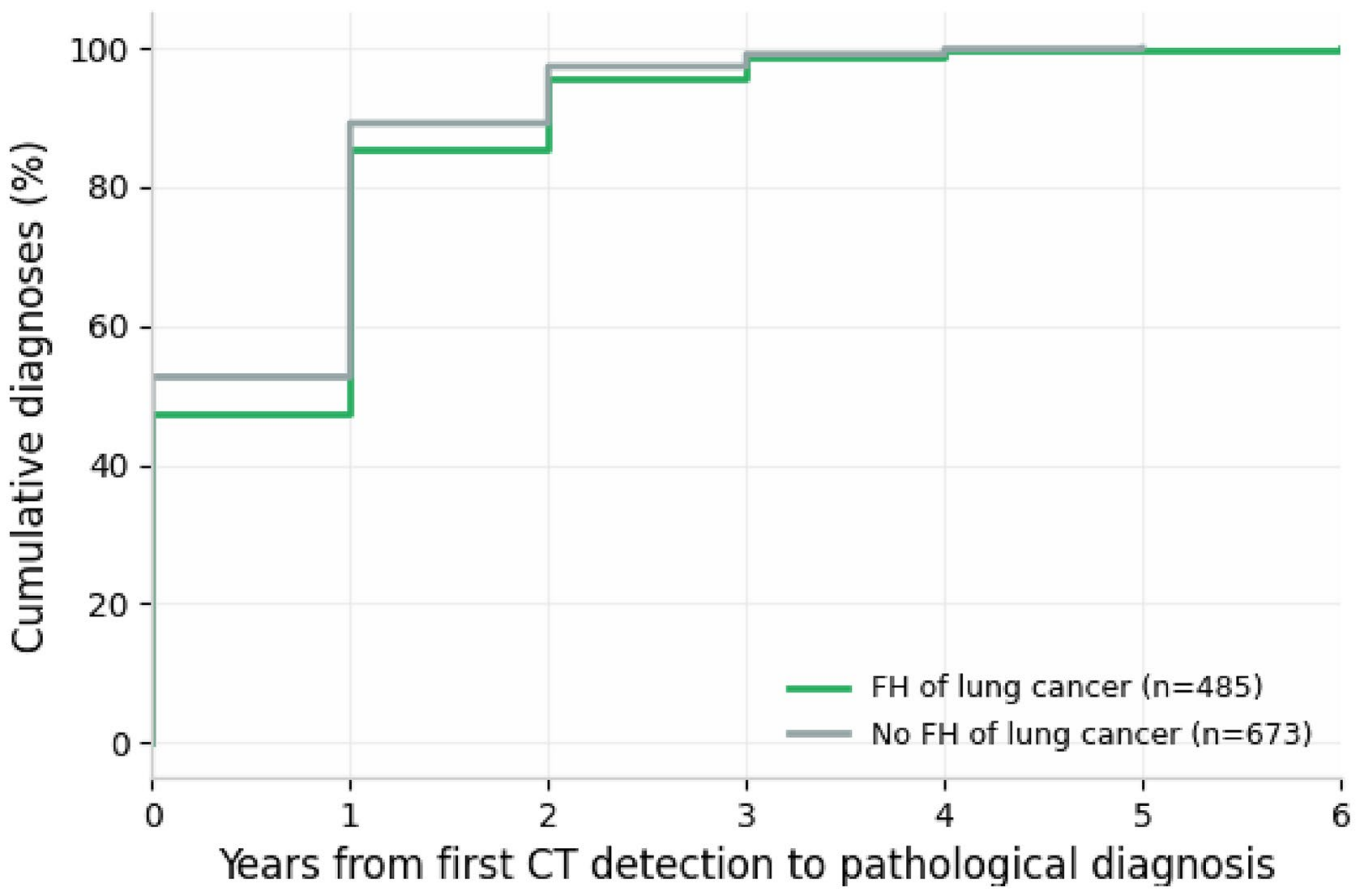
Time from first CT detection to pathological diagnosis of lung cancer, by family history of lung cancer (Mann–Whitney *p* = 0.035).

Among lung cancer cases, the interval from first CT detection to pathological diagnosis did not indicate earlier diagnosis in patients with family history of lung cancer: the median interval was 1.0 year (IQR 0–1) in patients with family history versus 0.0 years (IQR 0–1) in those without (Mann–Whitney *p* = 0.035), and 47.2% versus 52.6% of patients, respectively, were diagnosed at baseline (Figure 6). The proportion of stage IA disease did not differ significantly between the two groups (93.6% vs. 92.8%, *p* = 0.411; Figure 4D). Thus, we found no evidence that patients with a family history were diagnosed earlier or at an earlier stage, and the inverse adjusted association described above cannot be attributed to earlier detection in this cohort.

### 3.8 Pathology and stage distribution

The pathological and stage distributions of the 1,158 lung cancer cases are shown in Tables 2, 3. Adenocarcinoma accounted for 95.3% of cases, and 92.2% of patients were diagnosed at stage IA, reflecting the predominance of early-stage, screen-detected disease in this nodule-based cohort.

## 4 Discussion

### 4.1 Principal findings

In this retrospective cohort of 3,850 Chinese patients evaluated for pulmonary nodules, family history of lung cancer was not positively associated with the probability of malignancy in univariate analyses, and both family history of lung cancer and family history of non-lung cancer showed inverse associations after multivariable adjustment. Radiological features—nodule size, lobulation, spiculation, vacuole sign, and nodule type—were by far the strongest predictors of malignancy, increasing model discrimination from an AUC of 0.566 to 0.807. Female sex was independently associated with malignancy, whereas smoking was not, reflecting the predominance of never-smoking, early-stage lung adenocarcinoma in this cohort. Importantly, patients with a family history were not diagnosed earlier or at an earlier stage than those without.

### 4.2 Interpreting the inverse association: selection, referral, and surveillance-related bias

The inverse adjusted association between family history and malignancy should not be interpreted as evidence of biological protection; family history is a well-established risk factor for lung cancer in population-based studies (4, 5, 10). Several features of our cohort offer more plausible, non-causal explanations, which we present as hypotheses because screening intensity and prior imaging history were not directly measured.

First, referral and selection bias are likely. Individuals with a family history of cancer may be more health-aware, more likely to undergo chest CT for minor symptoms or health check-ups, and more likely to be referred to a tertiary center for evaluation of small or indolent nodules. This would enrich the family-history group with non-malignant nodules and dilute the proportion of malignancy. Second, surveillance-related bias may operate in the same direction: a lower threshold for repeated imaging and for biopsy or surgical referral among patients with a family history increases the detection of benign and indolent lesions. Third, the two family-history variables are correlated, and their mutual adjustment amplified the inverse estimates; in a selected cohort, such correlated exposure patterns are themselves shaped by health-seeking behavior. Analogous phenomena are well documented in other screening settings, where family history is associated with increased use of diagnostic procedures and increased detection of indolent disease (14, 15).

Notably, our data argue against an earlier-detection explanation: patients with a family history were not diagnosed more rapidly and did not present more frequently with stage IA disease. Because the outcome was the presence or absence of malignancy rather than post-diagnosis survival, the concept of lead-time bias is not directly applicable here; selection, referral, and surveillance-related biases are the more appropriate frameworks. These hypotheses require testing in prospective cohorts in which screening intensity and referral pathways are explicitly recorded.

### 4.3 Radiological features as the primary basis for nodule management

The most robust positive findings of this study concern imaging. Nodule size (aOR 1.21 per mm), lobulation (aOR 2.32), spiculation (aOR 2.08), vacuole sign (aOR 1.80), and mGGO morphology (aOR 1.26 versus pGGO) were strong independent predictors of malignancy, and adding these features to clinical variables raised the AUC from 0.566 to 0.807. These results reinforce that clinical decision-making for patients with pulmonary nodules should be driven primarily by imaging findings, operationalized through validated tools such as the Brock University model and Lung-RADS, both of which assign dominant weight to nodule size and morphology (16, 17). In a meta-analysis of 52 studies including 85,558 patients, the Brock model achieved a pooled AUC of 0.796, although performance was lower in Asian populations and in subsolid nodules, supporting the development of ethnicity- and nodule-type-specific models (17). Machine-learning approaches using CT features have reported similarly high discrimination (18).

By contrast, family history contributed little to discrimination in our cohort and showed directionally unstable estimates across models. Until the confounding and selection biases described above are addressed in prospective studies, family history should be interpreted with extreme caution when guiding the management of individual patients with pulmonary nodules and should not override imaging-based assessment.

### 4.4 Smoking and sex in this nodule-based cohort

Smoking was not significantly associated with malignancy after adjustment. This should not be read as evidence against the causal role of tobacco (19); rather, it reflects the composition of this cohort—79.9% never-smokers, only 8.9% heavy smokers, a mean age below 50 years, and 95.3% adenocarcinoma—in which smoking-related cancers presenting as larger symptomatic masses are underrepresented among patients evaluated for small nodules. In East Asian never-smokers, lung adenocarcinoma is driven predominantly by oncogenic driver mutations rather than tobacco carcinogens (20, 21). The independent association of female sex with malignancy (aOR 1.62) is likewise consistent with the well-described epidemiology of lung adenocarcinoma in Asian women (20, 22). Environmental exposures relevant to this population, such as cooking-oil fumes, were not systematically collected in this study and could not be evaluated (23, 24).

### 4.5 Family Cancer types: shared susceptibility versus shared environment

Among relatives of lung cancer patients, lung cancer was the most common reported malignancy (38.5% of events), consistent with familial aggregation through both genetic susceptibility and shared exposures (4, 5, 25). The clustering of gastrointestinal cancers (esophageal, gastric, colorectal, and pancreatic; together approximately 20% of events) and liver cancer (10.1%) plausibly reflects shared environmental and infectious exposures in Chinese populations, although shared DNA-repair susceptibility may also contribute (11, 13). These descriptive data cannot distinguish between these mechanisms.

### 4.6 Clinical implications

Our findings support the following cautious implications. First, imaging characteristics should remain the primary basis for the management of pulmonary nodules, and structured systems such as Lung-RADS and the Brock model should guide decisions about surveillance intervals and invasive procedures (16, 17). Second, family history may reasonably be elicited and considered during the assessment of patients with pulmonary nodules; however, the present study design is not sufficient to establish that family history should be incorporated into lung cancer screening protocols, and its independent contribution should be validated in prospective cohorts with standardized screening and referral procedures. Third, in line with previous studies, genetic counseling and germline testing may be discussed with selected patients who have strong familial clustering, early-onset disease, or multiple primary cancers (8, 26), although no genetic testing was performed in the present cohort.

### 4.7 Strengths and limitations

Strengths of this study include the large sample size, pathology-confirmed case definition, systematic recording of first- versus second-degree family history, and the use of sequential models that make the adjustment process transparent.

Several limitations must be emphasized. First, the retrospective, single-center, clinic-based design is subject to selection and referral bias, and the cohort is not representative of the general population or of a standardized screening program. Second, family history was self-reported and not verified against medical records or cancer registries, introducing potential recall and reporting bias, particularly for second-degree relatives and non-lung cancers. Third, screening intensity, prior imaging history, and referral pathways were not measured, so the biases proposed to explain the inverse associations remain hypotheses. Fourth, most controls were followed clinically without pathological confirmation; although this reflects real-world nodule management, residual misclassification of indolent malignant lesions cannot be excluded. Fifth, we did not collect detailed data on cooking-fume exposure, secondhand smoke, or ambient air pollution, and no genetic testing was performed, precluding direct assessment of germline variants or polygenic risk.

### 4.8 Future directions

Prospective cohorts with standardized low-dose CT protocols, verified family history, and recorded screening intensity are needed to separate genuine genetic risk from selection-related artifacts. Integration of validated polygenic risk scores with imaging-based models (27), and studies of gene–environment interactions in never-smoking Asian women, may further improve risk stratification (28–30).

## 5 Conclusion

In this large clinic-based cohort of Chinese patients evaluated for pulmonary nodules, family history of lung or non-lung cancer was not positively associated with the probability of malignancy, and the inverse adjusted associations are most plausibly explained by referral- and surveillance-related selection rather than biological protection. Radiological features—nodule size, lobulation, spiculation, vacuole sign, and nodule type—were the strongest predictors of malignancy and should remain the primary basis for nodule management. Family history may be considered during the assessment of patients with pulmonary nodules, but its independent contribution requires validation in prospective cohorts with standardized screening and referral procedures.

## Data Availability

The original contributions presented in the study are included in the article/Supplementary material, further inquiries can be directed to the corresponding author.
